# In Silico Prediction and Selection of Target Sequences in the SARS-CoV-2 RNA Genome for an Antiviral Attack

**DOI:** 10.3390/v14020385

**Published:** 2022-02-14

**Authors:** Mouraya Hussein, Zaria Andrade dos Ramos, Ben Berkhout, Elena Herrera-Carrillo

**Affiliations:** Laboratory of Experimental Virology, Department of Medical Microbiology, Amsterdam University Medical Centers, Academic Medical Center, University of Amsterdam, 1105 AZ Amsterdam, The Netherlands; m.hussein@amsterdamumc.nl (M.H.); z.s.deandradedosramos@amsterdamumc.nl (Z.A.d.R.)

**Keywords:** COVID-19, SARS-CoV-2 genome, CRISPR-Cas13d

## Abstract

The SARS-CoV-2 pandemic has urged the development of protective vaccines and the search for specific antiviral drugs. The modern molecular biology tools provides alternative methods, such as CRISPR-Cas and RNA interference, that can be adapted as antiviral approaches, and contribute to this search. The unique CRISPR-Cas13d system, with the small crRNA guide molecule, mediates a sequence-specific attack on RNA, and can be developed as an anti-coronavirus strategy. We analyzed the SARS-CoV-2 genome to localize the hypothetically best crRNA-annealing sites of 23 nucleotides based on our extensive expertise with sequence-specific antiviral strategies. We considered target sites of which the sequence is well-conserved among SARS-CoV-2 isolates. As we should prepare for a potential future outbreak of related viruses, we screened for targets that are conserved between SARS-CoV-2 and SARS-CoV. To further broaden the search, we screened for targets that are conserved between SARS-CoV-2 and the more distantly related MERS-CoV, as well as the four other human coronaviruses (OC43, 229E, NL63, HKU1). Finally, we performed a search for pan-corona target sequences that are conserved among all these coronaviruses, including the new Omicron variant, that are able to replicate in humans. This survey may contribute to the design of effective, safe, and escape-proof antiviral strategies to prepare for future pandemics.

## 1. Introduction

The severe acute respiratory syndrome coronavirus 2 (SARS-CoV-2) is the etiological agent of coronavirus induced disease 19 (COVID-19), which first became apparent in December 2019 in Wuhan, China, and subsequently caused the ongoing pandemic [1,2,3]. As of 3 January 2022, this zoonotic coronavirus has spread to 219 countries, with more than 290 million human cases, of which almost 5.5 million have died of atypical pneumonia. Phylogenetic analysis revealed that SARS-CoV-2 belongs to the beta-CoV group, and is most closely related to a SARS-like (SL) CoV found in bats in 2013 [4]. These two viruses share about 96% nucleotide sequence identity, suggesting that bats most likely served as the host reservoir for this SARS-CoV-2 pathogen with zoonotic capacity [4]. There may also have been an intermediate host that currently remains unknown. Since the beginning of the 21st century, two other beta-CoVs crossed the species barrier to cause deadly pneumonia in humans: severe acute respiratory syndrome coronavirus (SARS-CoV) [5,6], which emerged in China from bats and palm civets as an intermediate host in 2002 [7,8]; and the Middle-East respiratory syndrome (MERS) coronavirus, which continues to spill over from dromedary camels to humans in the Arabian Peninsula since 2012, but without efficient human-to-human transmission [9]. In addition to these highly pathogenic zoonotic coronaviruses, four low-pathogenicity coronaviruses are endemic in humans: the alpha-CoVs, HCoV-229E and HCoV-NL63, which also likely originated from bats [10,11,12,13,14,15]; and the beta-CoVs, HCoV-OC43 and HcoV-HKU1, which may originate from rodents [11,12,16,17,18]. To date, the U.S. Food and Drug Administration (FDA) and the European Medicines Agency (EMA) have approved one drug treatment for COVID-19 (Remdesivir), and have authorized others for emergency use. A rigorous search is still ongoing to identify new potent SARS-CoV-2 inhibitors, and repurpose available compounds [19,20,21,22,23]. To date, three vaccines have been approved against SARS-CoV-2 by the FDA and EMA (Pfizer-BioNTech, Moderna, and Johnson & Johnson’s Janssen), and a fourth one has been approved by EMA (AstraZeneca), but the SARS-CoV-2 pandemic remains a public health emergency. Here, we discuss alternative antiviral approaches that rely on a sequence-specific attack on the plus-strand viral RNA genome, the minus-strand RNA replication intermediates, or the many plus-strand mRNA species.

**Figure 1:**
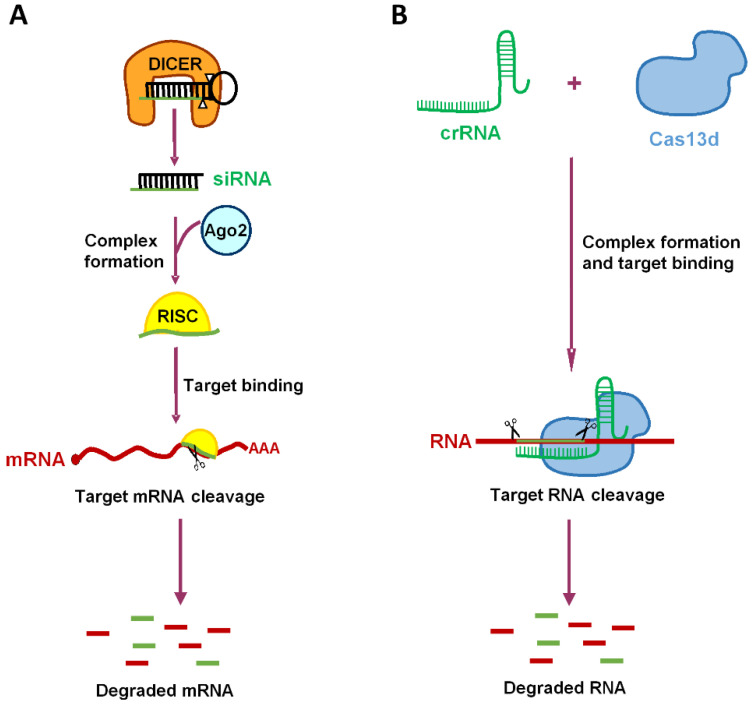
Comparison of the RNAi and CRISPR-Cas pathways. (**A**) shRNAs are expressed in the nucleus, and transported to the cytoplasm, where they are processed by Dicer into mature siRNA duplexes. siRNA duplexes are subsequently incorporated into the RNA-induced silencing complex (RISC), and one strand of the duplex directs this complex toward complementary mRNA targets that are inactivated by cleavage. (**B**) CRISPR-Cas13d activity in mammalian cells requires the expression of a CRISPR-RNA (crRNA) and the Cas endonuclease to form a complex that targets complementary RNA. Complementary RNA targets are inactivated by Cas cleavage. RNA cleavage is mediated by two nuclease domains (HEPNs; shown as two scissors).

The two systems we will deal with are an RNA-targeting CRISPR-Cas variant and RNA interference (RNAi), but the results are also relevant for other nucleic-acid-targeting mechanisms, such as aptamers, ribozymes, and antisense approaches. All these methods rely on sequence-specificity, and thus, can in principle, be designed against any viral pathogen, including those that cause future zoonosis. There are important similarities between the RNA-targeting CRISPR-Cas and RNAi mechanisms: both are mediated by small noncoding RNAs, and both possess the ability to knockdown gene expression at the post-transcriptional level (Figure 1). The RNAi system is present in all eukaryotic cells, and can be programmed by expression of man-made antiviral short hairpin RNA (shRNAs) [24,25,26,27,28]. However, we will put most emphasis on the clustered regularly interspaced short palindromic repeats (CRISPR)-Cas systems, which have provided scientists with a powerful and versatile gene-editing tool. Originally discovered as part of an immune system of archaea and bacteria [29,30,31], these systems have been optimized for use in human cells, and can now be programmed to target any genomic DNA sequence of interest [32,33,34,35,36]. CRISPR systems with different properties have been developed. We focus on the novel Cas13d nuclease because it selectively cleaves single-stranded RNAs that are complementary to the designed CRISPR-associated RNA (crRNA), and because it allows for an RNA-attack anywhere in the cell (nucleus, cytosol and other subcellular compartments), whereas RNAi activity is restricted to the cytosol [37,38,39,40].

**Figure 2:**
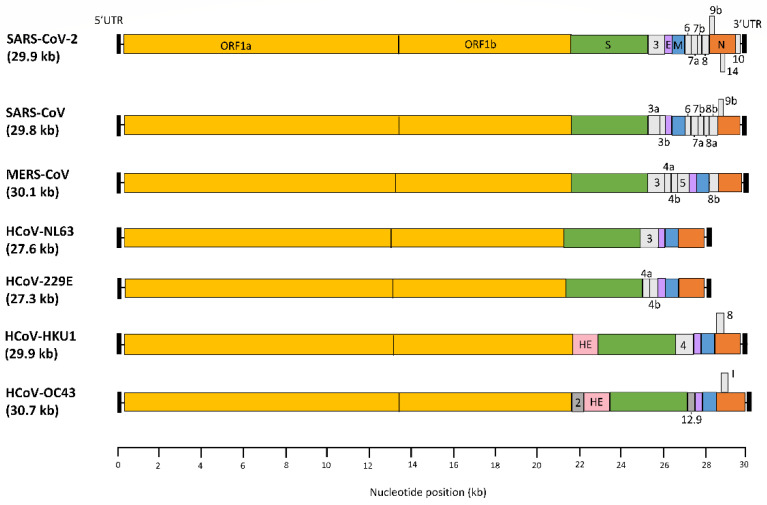
The RNA genomes of all seven human coronaviruses. The genome size is indicated behind the virus names (in kb), and relates to the corresponding reference strains (GenBank). All genomes have a 5′UTR and 3′UTR (black box), ORF1a/b (yellow box) encoding polyprotein 1a and 1ab, spike (S) gene (green box), envelop (E) gene (purple box), membrane (M) gene (blue box), and nucleocapsid (N) gene (orange box). Hemagglutinin esterase (HE) is an additional structural gene of HCoV-HKU1 and HCoV-OC43 (pink box). HCoV-OC43 encodes additional non-structural genes (dark grey boxes): 2, 12.9, and I. Accessory genes vary from virus to virus, and are shown in the light grey boxes.

The genome of coronaviruses is a single-stranded plus-sense RNA (+ssRNA) of ~27–31 kilobases with a 5′-cap structure and 3′-poly-A tail (Figure 2). Two thirds of the genomic RNA is used to encode the polyprotein 1a/1ab (pp1a/pp1ab), which is proteolytically cleaved by the viral papain-like protease (nsp3) and chymotrypsin-like protease (nsp5) into 16 non-structural proteins (nsps), designated nsp1–16, that are well conserved among the coronaviruses. The remaining portion of the genome encodes open reading frames (ORFs) for the structural proteins, including spike (S), envelope (E), membrane (M), and nucleoprotein (N), that are common to all coronaviruses. In addition, coronaviruses encode a variable number of accessory proteins that are translated from sub-genomic mRNAs. The SARS-CoV-2 accessory functions are ORF/protein 3, 6, 7a, 7b, 8 and 9b, 10, and 14 [18,41,42].

**Figure 3:**
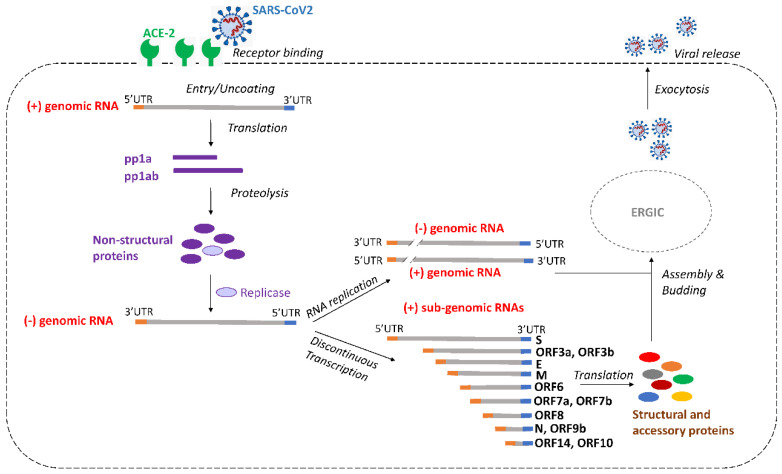
Schematic of the SARS-CoV-2 replication cycle. Upon virus binding to the ACE-2 receptor, plus-strand genomic RNA is released in the cytoplasm, and subsequently translated into the two polyproteins pp1a and pp1ab, with expression of the latter depending on a programmed −1 ribosomal frameshift at the ORF1a/ORF1b overlap region. Then, pp1a and pp1ab are proteolytically cleaved to generate 16 non-structural proteins (nsps). Several of these proteins form the replication complex that drives the synthesis of minus-strand genomic RNA, which, in turn, is copied into new plus-strand genomic RNAs that can be packaged in new virions. Discontinuous transcription generates a set of 3′ co-terminal sub-genomic mRNAs with an identical 5′UTR (shown in orange) and 3′UTR (shown in blue). The sub-genomic RNAs are translated into structural and accessory proteins that will form the new virions. Assembly and budding of the virions take place at the ER-Golgi intermediate compartment (ERGIC). Nascent virions are released from the cell via exocytosis.

Coronaviruses release the +ssRNA genome into the cytoplasm of infected cells to act as mRNA for translation of the polyproteins pp1a and pp1ab (Figure 3). The polyproteins are proteolytically processed into the different nsp functions. Some proteins form the replication/transcription complex that associates with double membrane vesicles in perinuclear regions [43,44]. A full-length complementary minus-strand RNA is synthetized, which is used as template for the production of new plus-strand RNA molecules, both full-length RNA genomes (genome replication), and subgenomic mRNAs via an orchestrated discontinuous transcription process during minus-strand synthesis that produces a set of 3′ co-terminal sub-genomic mRNAs with an identical 5′ leader sequence (Figure 3) [45,46]. Thus, the genomic plus-strand RNA genome and all plus-strand sub-genomic mRNAs share an identical 5′UTR (untranslated region) and an identical 3′UTR, making these regions optimal targets for an antiviral attack. Next, the translated structural proteins assemble into the nucleocapsid with a viral envelope at the endoplasmic reticulum (ER)–Golgi intermediate compartment (ERGIC), followed by exocytosis-mediated release of the nascent virion from the infected cell.

We set out to find optimal target sequences in the SARS-CoV-2 RNA genome for a Cas13d or RNAi attack. We first analyzed conserved viral RNA targets for the optimized and miniaturized Cas13d (CasRx) system [38,47]. The rationale for targeting conserved viral sequences is two-fold. The first and logical reason is that we want to target as many viral isolates as possible. The second less obvious reason is based on previous virus escape studies with the human immunodeficiency virus (HIV). We reported that there is less evolutionary freedom in conserved targets because mutations in these sites will cause a partial or complete virus replication-defect, thus restricting the potential for viral escape [48]. We first analyzed the genome of different SARS-CoV-2 isolates for highly conserved sequences, then broadened the survey to the related SARS-CoV RNA genome, the more divergent MERS-CoV virus, and the four endemic, but less pathogenic, “common cold” coronaviruses. This survey may help other researchers to quickly identify candidate target regions in the SARS-CoV-2 RNA genome for diverse antiviral approaches.

## 2. Materials and Methods

### 2.1. SARS-CoV-2 Genome Sequence Alignments

The sequence of the SARS-CoV-2 Wuhan Hu-1 RNA reference genome (MN908947) was aligned with 31,575 SARS-CoV-2 genome sequences available in the GISAID database, of which 28,294 represent “variants of concern” (VOC) named Alpha, Beta, Gamma, Delta, Lambda, Mu, and Omicron (https://www.gisaid.org/, accessed on 8 December 2021, Supplemental Table S1) [3]. In addition, SARS-CoV-2 sequences were aligned with the complete genomes of other human coronaviruses, ranging from closely to distantly related pathogens: SARS-CoV (260 strains), MERS-CoV (5 strains), HCoV-229E (24 strains), HCoV-NL63 (53 strains), HCoV-OC43 (168 strains), and HCoV-HKU1 (29 strains) (Supplemental Table S2). The multiple alignment program MAFFT (version 7, Osaka University, Osaka, Japan) was used to align the input data set [49]. All sequences were aligned to the reference strain MN908947 to avoid overloading of the software, and visualized using Bioedit 7.2.5. The BLASTN program was used to search the sequence databases with a specific sequence query.

### 2.2. Design of Antiviral crRNAs for a Cas13d Nuclease-Mediated Attack on SARS-CoV-2

Cas13d employs customizable crRNAs that directs the Cas13d protein to specific RNA molecules for targeted RNA degradation [50]. We ran a computational model (R script) developed by Wessels et al. [51] along the MN908947 reference genome (+ssRNA) to identify 23-nt crRNAs with high predicted efficacy. Next, Bioedit was used to identify the most conserved crRNA target sequences among different SARS-CoV-2 variants and other human coronaviruses. The Shannon entropy of the aligned sequences was calculated as a measure of the genetic variability per nucleotide position [52,53].

### 2.3. Design of Antiviral siRNA/shRNAs for an RNAi-Mediated Attack on SARS-CoV-2

There are different algorithms to identify optimal small interfering RNAs (siRNAs) [54,55,56,57,58,59,60]. Synthetic siRNA duplexes of ~21-nt complementary RNA strands with 2-nt 3′-overhangs can be designed perfectly complementary to the target mRNA to induce site-specific cleavage by the cellular Ago2 endonuclease. Alternatively, gene cassettes can be constructed to express short hairpin RNAs (shRNAs) that are intracellularly processed by Dicer into active siRNAs. The design of shRNAs remains largely an empirical process. A conventional shRNA consists of a 21-base-pair stem, a loop of at least 5-nt, and 3′-terminal dinucleotide overhang. We reported an alternative shRNA design, called AgoshRNA, with a shorter stem (17/19 base-pair) and small loop (3/5-nt) that triggers an alternative processing route [61,62]. We focused on this novel AgoshRNA design in this analysis because the target is shorter (19 versus 21 nt for regular shRNAs), which will yield more candidate viral targets with an absolutely conserved sequence [63,64,65,66,67,68,69]. For simplicity, we will collectively refer to both the original shRNA and new AgoshRNA design as RNAi inhibitors.

## 3. Results

### 3.1. Design of Optimal crRNAs for a Cas13d Nuclease Attack on SARS-CoV-2 RNA

**Figure 4:**
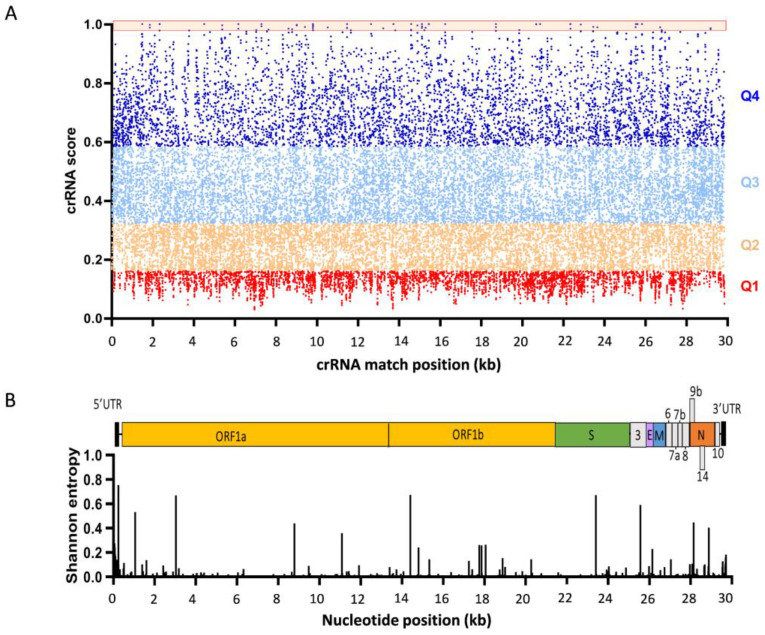
Design of optimal Cas13d crRNAs against SARS-CoV-2 RNA. (**A**). Score distribution of crRNAs along the SARS-CoV-2 RNA genome (MN908947). crRNA guide score is assigned to each crRNA along the complete SARS-CoV-2 genome (0–30 kb). Guide scores range from 0 to 1, with higher scores being indicative for higher predicted knockdown efficacy. The crRNAs are grouped in targeting efficacy quartiles Q1–Q4, with Q4 representing the best guide RNAs (guide score closer to 1). The top crRNAs with the maximal guide score of 1 are marked in the top orange box. (**B**). SARS-CoV-2 RNA genome diversity. The Shannon entropy along the RNA genome varies from 0 to 1, where lower genetic diversity gives values closer to 0.

The R script of Wessels et al. to design optimal crRNA molecules for the Cas13d nuclease was run along the RNA genome of 29,903 nt of the reference SARS-CoV-2 strain MN908947 [51]. The script generates all possible 23-nt crRNAs, and collects several features to predict the crRNA efficiency: crRNA intramolecular folding energy, crRNA—target RNA hybridization energy, and the target RNA context including accessibility [51]. The script removes crRNAs with homopolymer sequences of five or more Ts that will trigger premature termination of Pol III transcription of the transgene. Homopolymeric runs of six or more As, Cs, or Gs were also avoided because this may cause difficulties during oligonucleotide synthesis. This yielded a collection of 28,749 candidate crRNA targets of 23-nt, named after the position on the plus-strand RNA genome, e.g., crRNA1399 (Supplemental Table S3). The script predicts the on-target activity for Cas13d-mediated RNA cleavage, and ranks the crRNAs accordingly. Guide scores range from 0 to 1, with higher values predicting higher knockdown efficiency. The crRNAs were split in four quartiles (Q1–Q4) based on the crRNA efficacy, with Q4 representing the top inhibitors [51]. Figure 4A depicts the distribution of the predicted crRNA activity along the SARS-CoV-2 RNA genome, withthe four quartiles marked in different colors. Some 18.1% of the crRNAs are positioned in the top quartile Q4; the distribution of the ones with a lower predicted efficacy is as follows: 35.9% crRNAs in Q3, 30.8% in Q2, and 15.2% in Q1. Only 41 crRNAs (0.14%) have the maximum guide score of 1 (marked in the top box in Q4 in Figure 4A). These top predicted crRNA targets are well distributed along the viral RNA genome, but several nsp genes and S and ORF3a received multiple hits (4× nsp3, 2× nsp4, 3× nsp6, 1× nsp8, 8× nsp12, 1× nsp13, 1× nsp14, 2× nsp16, 5× S, and 7× ORF3a). Notably, no top scoring crRNAs were detected for the 5′ and 3′UTRs [51].

A similar analysis can be performed for the viral minus-strand RNA genome. One could argue that targeting of the minus-strand RNA would be preferable because it is expressed at a 100-fold reduced level compared to the plus-strand RNA in virus-infected cells, thus constituting an “easier-to-neutralize” target [63]. On the other hand, this also means that most or even all minus-strand RNAs may be annealed to the excess plus-strands to form the double-strand RNA replication intermediate. The minus-strand RNA may thus not be accessible to the Cas13d endonuclease, which has a strong cleavage preference for single-stranded RNA [36]. We therefore focused the analysis on viral +ssRNA targets (genomic and sub-genomic).

### 3.2. Selection of Optimally Conserved Human Coronavirus Target Sequences

The routine use of CRISPR-Cas technology for genome modification does not usually have to consider genetic variation in the target sequence, such that crRNA design is complete at this stage, followed by experimental validation of the highest-scoring crRNAs. However, viruses have a remarkably high mutational capacity, which creates genetic diversity that facilitates rapid adaptation to new conditions (e.g., antiviral drug pressure, or a new host upon zoonotic transfer). We therefore set out to define the most conserved crRNA target sequences among different SARS-CoV-2 variants. To increase the breadth of this antiviral strategy, it would be even better if these targets are also conserved in the genomes of other human coronaviruses.

We aligned the sequence of 31,576 SARS-CoV-2 virus isolates by multiple sequence alignment, including VOCs Alpha, Beta, Gamma, Delta, Lambda, Mu, and Omicron (MAFFT software). The Shannon entropy was plotted as a measure of the genetic variability per nucleotide position (visualized with Bioedit in Figure 4B). The Shannon entropy ranges from 0 to 1, where values close to 0 represent low genetic diversity [52,53]. We identified 100 absolutely conserved SARS-CoV-2 target sequences (cumulative entropy of 0) in the viral +ssRNA genomes (Supplemental Table S4). All identified target sequences are absolutely conserved between the most recent VOC Omicron and all the other SARS-CoV-2 variants. In theory, it is beneficial to select crRNAs that can target both the genomic RNA and the many sub-genomic RNAs (mRNAs). For instance, targeting of the 5′UTR or 3′UTR may affect the incoming viral plus-strand RNA genome, and thus, block the initial step of viral genome replication, but it would also target all plus-strand mRNAs (Figure 3). However, analysis of the genetic variation in Figure 4B revealed that these UTR segments do not contain any absolutely conserved sequences of at least 23-nt. This may in fact come as a surprise because these segments contain important replication signals. We realize that this sequence variation may represent technical artefacts, e.g., due to increased PCR and/or sequencing errors towards the ends of the viral genome. Therefore, 5′ and 3′UTR sequences were excluded from further analysis.

### 3.3. Selection of the Most Active Predicted crRNAs against Conserved SARS-CoV-2 Sequences

**Figure 5:**
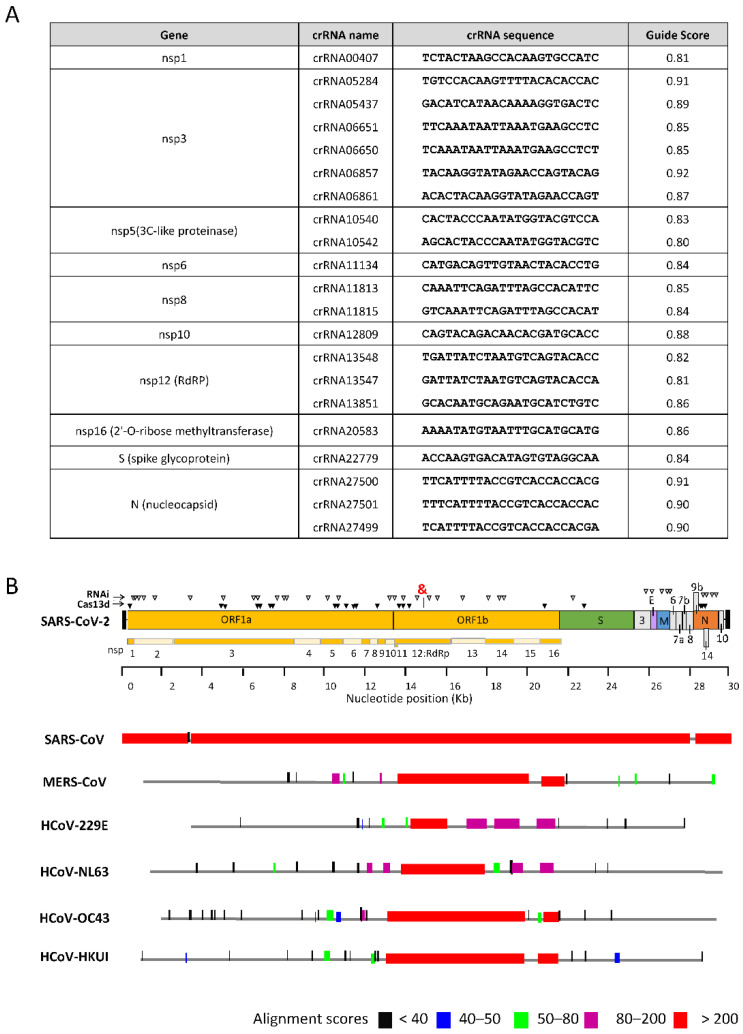
Candidate target regions in the RNA genomes of SARS-CoV-2 and all other human coronaviruses. (**A**) List of conserved crRNAs that target the SARS-CoV-2 genome with the best predicted knockdown efficacy (guide score ≥ 0.8). (**B**) Top: SARS-CoV-2 genome organization, including target regions for CRISPR-Cas13d and RNAi. Solid triangle: conserved Cas13d crRNA candidates. Open triangles: conserved RNAi candidates. RdRp: RNA-dependent RNA polymerase. &: the most conserved segment of 26-nt (15,283–15,314) among all human coronaviruses. Bottom: BLAST-mediated alignment of all other human coronavirus genomes (SARS-CoV, MERS-CoV, 229E, NL64, OC43, and HKU1), and color coding of the alignment scores. This score was computed by assigning a value to each aligned pair of bases, and counting these values over the length of the alignment.

We next calculated the predicted potency for Cas13d-mediated knockdown using the R script of Wessels for all possible 502 crRNAs of 23-nt for the conserved +ssRNA genome (Supplemental Table S4). The ranking and efficacy quartile Q1-Q4 are indicated with the best crRNAs in Q4. We identified 120 crRNAs (23.0%) in Q4, 310 (59.4%) in Q3, 38 (7.3%) in Q2, and 54 (10.3%) in Q1. In addition, we made a human-genome-wide search for similar sequences with the selected crRNA targets. Wessels et al. tested the tolerance to mismatches in the crRNA sequence, and demonstrated that increasing the number of mismatches in the crRNA to three mismatches abrogated target knockdown [51]. We therefore removed the potentially toxic candidates that are prone to cellular off-targeting (crRNAs with less than three mismatches with the human transcriptome). We next selected the top 20 crRNAs in Q4 with a predicted knockdown efficacy ≥0.8 and no potential off-target effect (Figure 5A and the targets on the viral genome are marked by solid triangles in Figure 5B). Of these, four crRNAs are special by targeting both the viral genomic RNA and several sub-genomic RNAs (S and N), and all other crRNAs target only the genomic RNA (nsp 1, 3, 5, 6, 8, 10, and 12).

### 3.4. Design of a crRNA Set to Prepare for the Next Pandemic

**Figure 6:**
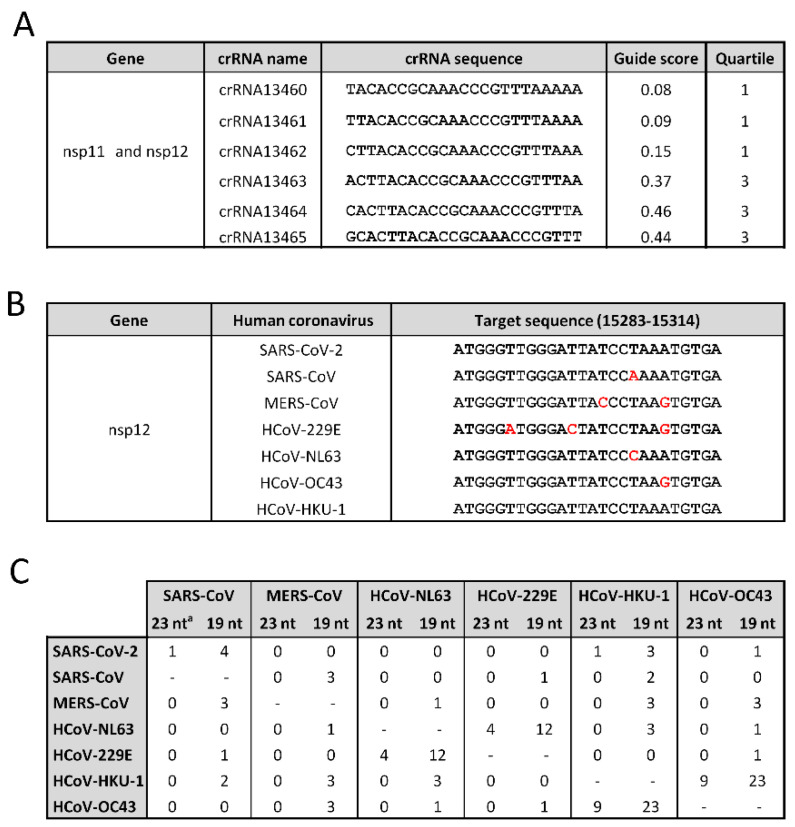
Conserved RNA target sequences of human coronaviruses. (**A**) crRNAs conserved among SARS-CoV-2 and SARS-CoV. (**B**) Highly conserved sequence in all human coronaviruses. Red letters represent mismatches in the selected target sequence. (**C**) The number of conserved Cas13d and RNAi target sequences shown per pairwise alignment of all human coronaviruses (a = target length), 23-nt, and 19-nt, respectively. Hyphen (-) represents cases of non-applicability of the pairwise alignment in the indicated positions.

As we should ideally prepare for future pandemic outbreaks of related coronaviruses, we next screened for candidate Cas13d targets that are conserved between the SARS-CoV-2 and SARS-CoV RNA genomes. The SARS-CoV-2 sequences were aligned with those of SARS-CoV, and conserved sequences were screened. This revealed one absolutely conserved target (entropy 0) with a length of 28-nt (TTTTTAAACGGGTTTGCGGTGTAAGTGC), which enables the design of six overlapping 23-nt crRNA molecules (Figure 6A). This prime target sequence encodes part of ORF1ab (13,460–13,487), in particular, the nsp11 protein of unknown function, and the N-terminal domain of nsp12 (RdRp, RNA-dependent RNA polymerase). As expected, this prime target overlaps with 1 of the 100 conserved hits in Supplemental Table S4 (13,457–13,502). We tested all possible 23-nt crRNAs for the predicted potency of Cas13d-mediated knockdown using the R script. Three crRNA candidates are in Q1 with poor knockdown efficacy ≤0.2, but some primary antiviral crRNA candidates are present in Q3 (crRNA13463, crRNA13464, and crRNA13465) with a predicted knockdown efficacy ≥0.3. The corresponding three crRNA targets overlap by 1 nt, which means that these crRNAs cannot be used in a combinatorial manner. Thus, it would seem important to select the best crRNA based on experimental validation of the antiviral activity in appropriate in vitro tests.

### 3.5. Towards Pan-Corona crRNAs

We next aligned the human SARS-CoV-2 RNA genome with that of all known human coronaviruses, including four relatively non-pathogenic “common cold” viruses. For this analysis, we used complete genome sequences of SARS-CoV, MERS-CoV, HCoV-229E, HCoV-NL63, HCoV-OC43, and HCoV-HKU1. We used the BLAST algorithm to identify stretches of sequence similarity, shown graphically as the alignment score in Figure 5B. BLAST generates bit scores that reflect the degree of similarity between hit and query sequences. An alignment score >200 indicates high similarity (genome regions marked in red). Several highly conserved segments with a score >200 for all seven coronaviruses were identified in the ORF1b gene. No absolutely conserved sequences (entropy 0) with a minimum length of 23-nt were identified. The most conserved segment of 26-nt was identified in the sequence encoding the essential RdRp in the ORF1b gene (15,283–15,314), marked with “&”in Figure 5B. Figure 6B depicts in red the few mismatches in this segment. Four overlapping crRNAs molecules of 23-nt can be designed with a number of mismatches that varies per coronavirus (none in HCoV-HKU-1, one in SARS-CoV/NL63/OC43, two in MERS-CoV, and three in HCoV-229E). Closer inspection reveals that this genetic variation represents silent codon changes in ORF1b that do not affect the encoded amino acids of the RdRp protein. The absence of any non-silent changes may cautiously suggest that such modifications are not allowed in this critical RdRp domain. As a consequence, the opportunity of viral escape from such crRNAs may be restricted; in theory, providing an extended therapeutic window.

As a second-best strategy, we analyzed sequence similarities between pairs of the seven human coronaviruses. We plotted the number of absolutely conserved targets in Figure 6C, and listed the corresponding sequences in Supplemental Table S5. For instance, we identified nine absolutely conserved sequences between HCoV-OC43 and HCoV-HKU1, and four between HCoV-229E and HCoV-NL63 (Figure 6C). These candidate targets are located in ORF1b encoding the nsp12 (RdRp), nsp13 (helicase), nsp14 (3′-to-5′ exonuclease), nsp16 (2′-O-ribose methyltransferase), and the gene encoding the spike (S) glycoprotein (Supplemental Table S5). All targets are obviously present in the full-length viral +RNA genome, but the S targets are also present in a single sub-genomic mRNA (sub-genomic RNA S).

No absolutely conserved crRNA target sequences were found between MERS and any of the other human coronaviruses. However, this analysis did reveal that a combination of just four crRNAs will suffice to target all known human coronaviruses (one crRNA each for the clusters SARS-CoV-2/SARS-CoV, MERS, HCoV-OC43/HCoV-HKU1, and HCoV-229E/HCoV-NL63 in Supplemental Table S5). As MERS is currently not spreading from human-to-human, this pan-corona set could be reduced to three crRNAs.

### 3.6. Selection of Conserved HCoV Targets for an RNAi Attack

We thus far focused on Cas13d as an antiviral mechanism, but the same principles for selecting the best viral target sequences hold for other sequence-specific antiviral tools. The RNAi mechanism can block the replication of a variety of viruses, including HIV, hepatitis C virus, and also SARS-CoV in cultured human cells [27,64,65,66,67,68,69,70,71]. The design of short hairpin RNAs (shRNAs) that are expressed from a transgene construct remains an empirical process, but several algorithms exist for the design of small interfering RNAs (siRNAs) [54,55,56,57,58,59,60,72]. In this theoretical exercise, we will consider the shRNA route. In fact, we will focus exclusively on the novel AgoshRNA design, which has several advantages over the traditional shRNA design [61,73]. Importantly, AgoshRNAs use a relatively small target sequence of 19-nt, which means that one can expect to find more conserved targets compared to approaches that use a longer target (e.g., 21-nt for shRNAs, and 23-nt for crRNAs). For simplicity, we will refer to the AgoshRNAs as RNAi inhibitors.

As for the crRNA design, it will be important to target sequences that are well conserved among virus isolates in order to obtain a broad-spectrum antiviral. Our analysis revealed more well-conserved candidate RNAi targets (430) than Cas13d targets (100) in the HCoV-SARS-2 RNA genome, consistent with the differential target length requirement (Figure 6C). These ideal RNAi targets are distributed over all viral genes (Supplemental Table S6). Next, we screened the candidate RNAi inhibitors for high predicted cleavage efficacy and low off-target effects using the siPRED and siSPOTR algorithms, respectively [56,57]. Some 430 RNAi candidates were identified with a predicted efficacy between 41 and 95% (Supplemental Table S6), and we subsequently removed the potentially toxic candidates that are prone to off-targeting. A potential off-targeting score (POTS) value below 52 was set as an arbitrary cut-off value [56]. Only 35 of the 430 RNAi candidates (8.1%) satisfy both predicted efficacy and off-target qualifications, and form the optimal set (indicated with open triangles in Figure 5B).

We next screened for RNAi inhibitors that are conserved between SARS-CoV-2 and SARS-CoV (Figure 6C). Four absolutely conserved RNAi target sequences were identified in the ORFs for nsp5, nsp8, nsp11/nsp12 (exclusively present in the full-length RNA genome), and E (also present in multiple sub-genomic RNAs). We listed these four primary candidate RNAi inhibitors of 19-nt in Supplemental Table S5.

We next searched for well-conserved target sequences that are present in the RNA genome of all seven human coronaviruses. No absolutely conserved target was identified. We next analyzed virus pairs, and plotted the number of absolutely conserved targets for these virus pairs (Figure 6C). The majority of these RNAi targets are located in ORF1a. As expected, we identified more candidate RNAi targets than Cas13d targets, which holds true for the analysis of virus pairs.

## 4. Discussion

The global impact of COVID-19 has been tremendous, and has triggered an intense international research effort to develop therapeutic strategies against SARS-CoV-2. Although this search has yielded some drug candidates, such as Veklury (remdesivir) [74], one should especially consider the breadth of such approaches, as new variants keep emerging, urging us to prepare for future pandemic outbreaks of related coronaviruses [60]. Despite the identification and repurposing of inhibitory small molecules, tools from the gene editing field offer a straightforward alternative that can be employed systematically. Prior research has demonstrated that both the RNAi and CRISPR-Cas13d systems can be converted into potent antiviral agents against a diversity of RNA viruses [27,64,65,66,67,68,69,70,71,75,76]. To facilitate the rapid development of such antivirals for SARS-CoV-2, we performed an initial in silico analysis to identify optimal target sequences in the SARS-CoV-2 RNA genome. We propose to target highly conserved SARS-CoV-2 genome sequences, preferentially those that are also conserved in the related SARS-CoV and other human coronaviruses. Targeting of both the viral genome and the many viral subgenomic mRNAs by RNAi or CRISPR-Cas13d should effectively degrade the templates for SARS-CoV-2 replication and viral protein expression. This may lead to a robust and durable restriction of virus replication, as was previously shown using RNAi in SARS-CoV [71].

We compared CRISPR-Cas13d and RNAi as antiviral mechanisms in silico. Both mechanisms are guided by small noncoding RNAs that dictate the sequence-specific attack. A major difference between these systems is their biological origin. All components of the RNAi mechanism (e.g., DICER, Argonaute 2) are present in all human cells to regulate cellular gene expression at the post-transcriptional level. Induction of an antiviral RNAi response only requires the expression of a novel guide RNA. By contrast, CRISPR-Cas systems originate from bacteria, where they provide a sequence-specific defense mechanism against the genomes of invading bacteriophages. This means that the complete CRISPR-Cas machinery (Cas endonuclease and crRNA) has to be introduced in human cells. In addition, a CRISPR-Cas-based therapy will be more immunogenic than RNAi that produces no non-human protein. However, RNAi also has some possible drawbacks. For instance, one has to be careful not to influence the cell physiology by saturation of the endogenous RNAi machinery that controls cellular gene expression [77,78]. We previously discussed the respective advantages and disadvantages of these systems in the context of an HIV-1 gene therapy [79]. A major advantage of CRISPR-Cas is that the design of antiviral crRNAs is more robust than RNAi design, as most candidate crRNAs, when independent of their sequence, are usually active [24,79,80]. For instance, for HIV inhibition strategies, we reported that most guide RNA designs were fairly active, whereas a fair percentage of shRNAs were inactive [24,80]. One possible explanation for this difference is that the structure of the double-stranded DNA target for CRISPR-Cas is constant, whereas that of different RNAi targets is quite variable in terms of secondary and tertiary RNA structure, which may affect the efficiency [81]. The latter variable may also affect the RNA-targeting Cas13d system. A clear crRNA advantage is that these molecules do not require complex intracellular processing steps, whereas shRNAs have complex sequence and structure requirements for optimal intracellular transport and enzymatic processing by Dicer and Ago2.

We included RNAi in this in silico analysis because the RNAi target is shorter than the CRISPR-Cas target (19 versus 23 nt), which resulted in more candidate targets. We specifically screened for viral target sequences that are well conserved in diverse SARS-CoV-2 isolates, and preferentially in the other human coronaviruses. We found more absolutely conserved RNAi than Cas13d targets in all human coronaviruses. Our analysis identified several highly-conserved regions in the ORF1b that will allow targeting of all known human coronavirus genomes and, possibly, also novel coronaviruses that may cause the epidemic or pandemic (Supplemental Table S6).

We identified the most conserved target sequences in the critical RdRp gene: target 15,283–15,314: ATGGGTTGGGATTATCCTAAATGTGA (Figure 6B). Moreover, sequence alignment indicates that the SARS-CoV-2 RdRp is almost identical to that of SARS-CoV (98% similarity, Figure 5). Thus, RdRp could be the target of choice for the design of effective and escape-proof antivirals due to its high sequence conservation. We identified the highest-scoring crRNAs considering crRNA features and target RNA context (Supplemental Table S4) [51], and selected those with highly conserved target sequences (Figure 5A).

We aligned all known human coronaviruses in a pairwise manner. This analysis revealed that a combination of just four crRNAs suffices to target all these coronaviruses (Supplemental Table S5). It is important to realize that this pan-corona crRNA set can be adjusted over time to keep track with the ongoing evolution of these pathogens. Virus evolution is usually a gradual process, but bigger jumps in sequence space are also possible. For instance, coronavirus genomes are subject to recombination events, thereby increasing their evolution capacity [41,82,83]. SARS-CoV-2 produced by humans could also establish a reservoir in a new animal host, adapt rapidly to the new environment, and then get reintroduced into the human population. This is not unthinkable, as SARS-CoV-2 already jumped into farmed minks in six countries [84,85,86]. Sequence analysis of the mink-derived viruses indicated that humans were the likely source of this human-to-animal zoonotic transmission.

Targeting the positive-sense viral genome and mRNAs to degrade both the viral templates for genome replication and translation is expected to robustly limit virus replication. One could also consider targeting the negative-sense RNA genome, which is present in much lower numbers than positive-sense RNA, and may thus be easier to suppress [63,87]. However, the negative-strand may have a relatively short half-life, and may be protected by the double membrane vesicles in which genome replication takes place [43,44]. In addition, the negative-strand RNA may not exist as naked RNA because of annealing to the more abundant positive-strands [63,87]. Cas13d preferentially cleaves unstructured targets, and the predicted RNA secondary structure of the target is negatively correlated with the knockdown efficiency in bacteria and mammalian cells [36]. Thus, naturally structured motifs in the coronavirus RNA should be avoided.

The relevance of a fast method for the in silico design of antivirals that target highly conserved viral genome sequences has recently become more apparent in the SARS-CoV-2 pandemic by the sudden appearance of the heavily mutated Omicron variant, but also in order to prepare for future outbreaks of yet unknown coronaviruses from ill-described animal reservoirs [88,89,90]. The most recently identified VOC Omicron raised concerns because it acquired some 30 mutations in the Spike gene, which could have major implications concerning virus replication and cell tropism, pathogenicity, vaccine efficacy, and resistance to antivirals [91,92,93,94,95]. Recent in vitro data by Wilhelm et al. indeed showed that the monoclonal antibodies Imdevimab and Casirivimab failed to neutralize Omicron [95,96].

Several previous studies presented detailed in silico analyses to select the best targets in the viral genome, but the current study supplements these available datasets, as our final selection of crRNAs and siRNAs is based on the complete genome analysis of all seven human coronaviruses, including all VOCs of SARS-CoV-2 that are known to date. The in silico studies by Abbott et al. and Wang et al. focus only on specific regions of the SARS-CoV-2 genome: the RdRp/Nucleocapsid and Spike genes [88,89]. We checked the performance of the previously designed crRNAs in our in silico platform. Some of the crRNA targets designed by Abbott. et al. are not conserved among the Delta and Mu variants, and none of the crRNA targets designed by Wang et al. are absolutely conserved in the Delta and Omicron variants [88,89]. On the other hand, siRNAs designed by Chowdhury et al. are well conserved among all SARS-CoV-2 VOC isolates known to date [90]. Abbott et al. described a set of 6 crRNAs that cover more than 90% of the human coronaviruses, but some 22 crRNAs were needed to cover all human coronaviruses. Instead, our analysis generated a list of 100 crRNAs against sequences that are highly conserved sequences among all 7 SARS-CoV-2 VOCs, and we identified a set of just 4 crRNAs that covers all known human coronaviruses.

Our in silico crRNA design strategy centered around the criterion of conservation of target sequences across all known human coronaviruses, including SARS-CoV-2 variants that recently emerged. The idea behind this strategy is that it will allow the development of an escape-proof antiviral platform because well-conserved viral genome sequences are known to allow less sequence variation and, thus, viral escape [48]. The repeated emergence of new SARS-CoV-2 variants underscores the pressing need to design such broadly active antivirals [91]. Recent studies by Ko et al. and Wu et al. emphasize the added value of our systematic approach, as they have identified possible therapeutic targets for the treatment of MERS-CoV infections which can be extended to SARS-CoV-2 [97,98]. Our systematically generated set of target regions in SARS-CoV-2 based on the criterion of sequence conservation can be used to contribute to the fast identification of antivirals for SARS-CoV-2. Moreover, our panels of 100 CRISPR-Cas13d and 430 RNAi targets remained perfectly conserved among Omicron isolates (Supplemental Tables S4 and S6). This result underscores the value of our systematic approach for the design of sequence-specific antivirals. In order to keep pace with ongoing virus evolution in the future, quick adaptation of the sequence of these antivirals will be possible.

## 5. Future Perspective

We plan to initiate a translational research line to develop a Cas13d-based antiviral therapy. The first step would be to experimentally validate these crRNA (and RNAi) designs in simple cell culture models. One could first test the knockdown efficiency on a simple luciferase reporter construct in transiently transfected cells. The best inhibitors could be selected for a subsequent test against the replicating virus. As SARS-CoV-2 can only be handled in a biosafety level 3 laboratory, one could consider the use of non-infectious replicons that contain all genes necessary for RNA replication and an exogenous reporter gene [99,100]. Inhibition of viral RNA replication can simply be measured as a reduction in reporter gene expression.

The second step is to avoid inhibitors that exhibit off-target effects and potential toxicity. For both Cas13d- and RNAi-based inhibitors, off-target cleavage of unrelated human RNAs has to be considered. Algorithms have been developed to predict such off-target effects, but the number of the potential off-targets varies widely among different algorithms, and it is questionable whether all off-target effects can be predicted [101,102,103]. A direct experimental comparison revealed significant off-target effects for RNAi, but none for Cas13d [104,105]. A direct comparison of the activity in human cells revealed that CRISPR-Cas13d technology is more efficient (80–95%) than RNAi (~70%) [104]. Therefore, based on better activity and reduced off-targeting, we would suggest CRISPR-Cas13d as the more promising choice for targeting the RNA genome of human coronaviruses. The crRNAs selected will need to be evaluated experimentally for off-target effects by using different techniques, such as the Competitive Cell Grow (CCG) assay and transcriptome RNA sequencing [106].

The third step is about efficient in vivo delivery of the selected crRNAs into the human lung epithelial cells in which SARS-CoV-2 replicates [107]. One could deliver Cas13d as a ribonucleoprotein complex (RNP) with crRNAs [108]. Another strategy would be to deliver the crRNA to airway epithelia by engineered amphiphilic peptides [109]. Cas13d and the crRNAs candidates could also be delivered as RNA within lipid nanoparticles or chemical polymers [109,110,111]. An inhalable CRISPR-Cas13d formulation could be prepared for administration in the lungs [112].

Our research was focused on finding optimal target sequences in the SARS-CoV-2 RNA genome for a therapeutic attack, but this work may also have relevance for viral diagnostics based on the CRISPR-Cas technology, in particular, the interaction between the crRNA and the viral RNA genome [113,114,115]. It seems fair to extend our current finding of optimal therapeutic target sites to candidate target sequences for viral diagnostics. Having the best coverage among circulating virus strains also means the best diagnostic value, as most virus isolates will be detected, including the VOCs and Omicron. Besides using the generated set of crRNAs for therapeutical purposes, they can be employed in novel detection methods based on CRISPR-Cas. For instance, FDA have already issued an emergency use authorization (EUA) for SHERLOCK and DETECTR assays for SARS-CoV-2 detection [116,117]. As demonstrated by the pairwise alignments among all seven human coronaviruses, our selected panel would be valid to be used for the detection of not only SARS-CoV-2, but also other human coronaviruses (Figure 6).

## Data Availability

Not applicable.

## Supplementary Materials

The following supporting information can be downloaded at: https://www.mdpi.com/article/10.3390/v14020385/s1, Table S1. SARS-CoV-2 genome sequences available in the GISAID database, Table S2: Genomes of other human coronaviruses, Table S3: Design of crRNAs for the Cas13d nuclease against SARS-CoV-2, Table S4: Design of crRNAs for the Cas13d-mediated knockdown against SARS-CoV-2 genome, Table S5: Sequence similarities between pairs of the seven human coronaviruses, Table S6: Conserved SARS-CoV-2 target sequences of a minimum length of 19 nucleotides.
